# Feasibility and acceptability of an online guided self-determination program to improve diabetes self-management in young adults

**DOI:** 10.1177/20552076231167008

**Published:** 2023-03-30

**Authors:** Bodil Rasmussen, Karen Wynter, Peter S Hamblin, Christine Rodda, Cheryl Steele, Sara Holton, Vibeke Zoffmann, Judy Currey

**Affiliations:** 1 School of Nursing and Midwifery, Centre for Quality and Patient Safety Research in the Institute for Health Transformation, 2104 Deakin University, Geelong, Australia; 2 The Centre for Quality and Patient Safety Research in the Institute of Health Transformation – Western Health Partnership, 95317 Western Health, St Albans, Australia; 3 Faculty of Health and Medical Sciences, 4321 University of Copenhagen, Copenhagen, Denmark; 4 Faculty of Health Sciences, University of Southern Denmark and Steno Diabetes Center, Odense, Denmark; 5 Endocrinology & Diabetes Department, 95317 Western Health, St Albans, Australia; 6 Institute for Health Transformation, Faculty of Health, 2104 Deakin University, Burwood, Australia; 7 Western Health, University of Melbourne, St Albans, Australia; 8 Western Health, Sunshine Hospital, St Albans, Australia; 9 Department of Public Health, 4321 University of Copenhagen, Copenhagen K, Denmark; 10 The Interdisciplinary Research Unit of Women's, Children's and Families’ Health, Juliane Marie Centre: Copenhagen University Hospital, Rigshospitalet, Copenhagen, Denmark

**Keywords:** Diabetes, young adults, guided self-determination, self-management, online, health communication, Australia

## Abstract

**Objective:**

Evaluate the feasibility and acceptability of an online guided self-determination (GSD) program to improve diabetes self-management skills among young adults with type 1 diabetes (YAD).

**Methods:**

An online program comprising seven structured interactive conversations was designed. A pre- and post- interventional study used a sequential, two-phase multiple method design. Phase one comprised a training program for diabetes educators (DEs). In Phase two YAD participated in program and completed pre- and post-surveys assessing motivation to self-manage, perceived competence in diabetes and communication with DEs. Both YAD and DEs provided a program evaluation.

**Results:**

The online GSD program was acceptable, feasible and effective in improving autonomous motivation in self-management and communication with DEs. Easy access and program flexibility were highly appreciated by both participant groups and perceived to assist YAD to stay motivated.

**Conclusion:**

The program had a significant impact on the diabetes self-management of YAD and was a feasible and acceptable way to engage and communicate with DEs. The GSD platform contributes to age appropriate and person-centred diabetes self-management. It can potentially reach geographically distanced populations, or with social circumstances or other barriers impeding in-person service provision.

## Introduction

Supporting optimal diabetes mellitus (DM) management in young people, especially those aged 18 to 25 years can be challenging for many reasons^1,2^ including routine clinic visits being typically held during office hours, potentially resulting in missed tertiary education classes or work. Inadequately managed DM resulting in poor glycaemic control is associated with increased risk of developing complications including visual loss, amputation, neuropathy, end stage renal disease, cardiovascular disease (CVD), infections and cognitive impairment.^3^ Living with diabetes requires lifelong self-management to achieve optimal blood glucose levels and well-being and reduce the risk of developing complications. Providing education about self-management is critical in diabetes care. Three main pillars of diabetes care and management have been identified: patient empowerment, self-management education and lifestyle modification. Specific support strategies for young people with diabetes while recognising their changing life-stage need to be combined with other behavioural strategies to motivate them to effectively manage their diabetes.^4^

Diabetes self-management depends on the individual's motivation and autonomy, as motivation is an important conceptual variable in diabetes care.^5^ As self-management of diabetes is ongoing, motivation is a process rather than related to a specific goal.^6^ Self-determination theory, provides a useful, process-based framework, and addresses the basic human need for competence, while also considering individuals’ needs for ownership and internalisation of their behaviour.^5,7,8^

Different types of motivation to self-manage diabetes are found to be related to different outcomes^9,10^: a motivation seen in people who “experience no meaningful relation between what they are doing and themselves” and controlled motivation is identified in people who wish to obtain a reward or avoid negative consequences are both connected with poorer health outcomes.^9,10^ In contrast, autonomous motivation, is identified in people whose behaviour is positively endorsed and valued by them is associated with positive health, behavioural and psychological outcomes, such as to follow medication advice and weight-loss regimens.^6,11^

Current support interventions to achieve optimal diabetes self-management thus tend to support peoples’ autonomous motivation.^12^ Guided self-determination (GSD) is a person-centred reflection and problem-solving method based on self-determination theory^13,14^ has been shown to improve life skills based on self-determination and problem-solving strategies in YAD.^1,15^ Life skills are defined as “those personal, social, cognitive and physical skills that enable people to control and direct their lives, and to develop the capacity to live with and produce change in their environment”.^12^ The GSD method provides educational and behavioural support for a person's own motivation and skill development in their diabetes self-management.^13^ People with diabetes are prompted by health professionals to systematically explore and express their personal difficulties and experiences with their condition through words and drawings on shared 4–8 worksheets (known as ‘conversations’). Discovering and expressing personal challenges and priorities related to their conditions enables people to discover their potential for change.^14^ The GSD method facilitates shared decision-making and improves health professionals’ advanced communication strategies.^16,17^

To date, the GSD program delivery with YAD has mainly been facilitated in-person, consequently geographical, travel, cost and time are barriers to in-person participation, especially in Australia where YAD may live vast distances from cities. Most young Australians have internet access and use it daily.^18^ Technology has also been found to be successful in supplementing healthcare through the provision of educational and motivational support.^19^ Accordingly, the research team converted the existing in-person GSD program for online access and facilitation, so YAD had access regardless of their geographical location.

The online GSD program has been tested for proof of concept in Australia by members of the research team^20,21^; the online format was a suitable, convenient and improved way to communicate and engage with YAD managing type 1 diabetes (T1D). In previous studies, YAD and participating diabetes educators found the online communication expedient, flexible and particularly suitable for the YAD who had lost motivation.^20,21^ The online GSD program used in the current study has been modified based on feedback from this proof-of-concept study.^20^

The main aim of this study was to assess feasibility, acceptability and efficacy of the online GSD program to improve self-management of T1D among young adults. The secondary aim was to investigate experiences of online GSD from the perspectives of both YAD and diabetes educators (DEs).

## Key abbreviations

GSD = Guided Self Determination

YAD = Young adults with Diabetes

T1D = Type 1 Diabetes

DE = Diabetes Educator

## Methods

### Design

**Figure 1.**
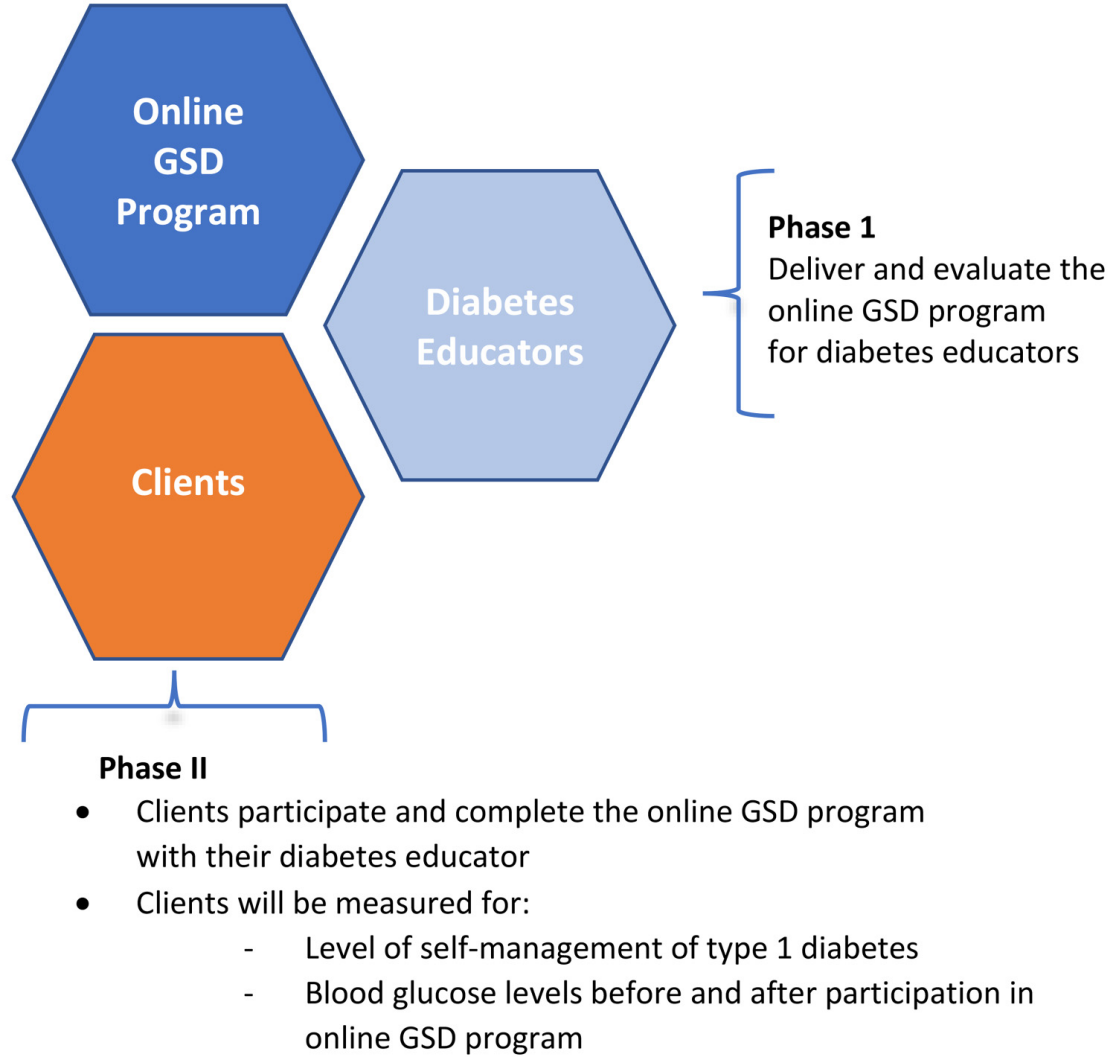
Illustration of the two phases of the study.

A pre- and post- interventional study using a sequential, two-phase multiple method design was applied (Figure 1).

Multiple-method research focuses on the use of multiple types of data from a singular paradigm to answer the research questions in one study.^22–25^ The first phase was the delivery of the training program in online GSD for DEs. The second phase was YAD participation in, and completion of, the GSD online program with a GSD-trained diabetes educator.

### Participants and recruitment

The DEs were eligible to participate if they had at least 5 years’ experience in diabetes care. Recruitment was through advertisements in social media and newsletters of the Australian Diabetes Educators Association (ADEA). A plain language statement and consent form was sent by e-mail to each DE who expressed an interest by contacting the research team. Written consent to participate was provided by each DE.

YAD were eligible to participate if they were aged 18–30 years, diagnosed withT1D, had access to mobile phone, iPad or computer and ability to provide self-measured blood glucose results.

They were recruited from (a) the Young Adult Diabetes Clinic at a metropolitan health service in Melbourne, Australia; (b) advertisements in the Victorian consumer diabetes organisation's social media, newsletters and website; (c) social media advertisements and posters at the university where research team members are employed. At the Young Adult Diabetes Clinic, YAD waiting for endocrinologist appointments was approached by a research assistant, who provided a flyer about the study and a plain language statement and consent form. YAD was invited to ask questions about the study, and if they were interested in participating, they signed their consent and kept their copy of the plain language statement and consent form. YAD who saw advertisements emailed the research team, then received an emailed plain language statement and consent form that they signed and returned to participate.

### Procedure and intervention

Training for DEs included online learning materials, and a one-and-a-half day, in-person, introductory workshop. The workshop aimed to provide DEs with an understanding of the theories (self-determination, empowerment and communication) that underpin the GSD program; the specific behavioural characteristics of older adolescents, in the 18–25-year-old age group; an opportunity to practice the three GSD-informed communication skills of active listening, mirroring and value clarification; and an introduction to and use of the online GSD platform. Completing training and receiving accreditation as a GSD diabetes educator required DEs to complete the online GSD program with two YADs, while being mentored by members of the research team and experienced GSD-trained DEs.

The GSD program for YAD consists of synchronous conversations, facilitated through the purpose-built GSD platform, between YAD and DEs at mutually convenient times. The conversations include worksheets which are completed by YAD in discussion with the DE, with some allocated activities completed separately by each YAD between conversations. The activities encourage reflection by the YAD. For example, the YAD is prompted to:

- Indicate significant events/turning points in their lives with diabetes on a timeline
- Draw/upload a photo or image to illustrate YAD's feeling of living with diabetes
- Fill out unfinished sentences to reflect on how YAD perceive themselves and why they manage diabetes in the way they do
- Use interactive circles to indicate how much diabetes take up time in their lives and how much they wish diabetes to take time and the differences were then discussed with DEs
- Fill out a model that underpins their values and helps to identify the most pertinent current challenge in their life with diabetes

The final two conversations focus on decision strategies to solve the identified challenge and any future steps in managing their life with diabetes. The online GSD consultations between DEs and YAD were conducted via Zoom or by telephone, according to the preference of the YAD. For each young adult, participation in the GSD online program included seven interactive conversations over 6 months.

### Outcomes

To meet the main study aim, online surveys with validated psychometric instruments were used before and three months after participation. To meet the secondary study aim, fixed-response and open-ended questions were included in the second, post-participation survey for YAD. An online survey including fixed-response and open-ended questions was used to assess acceptability from the perspectives of DEs. A focus group with DEs was facilitated to gather in-depth experiences of the program (Table 1).

**Table 1.** Components of the multiple method design assessing each study aim.

Aim: to assess	Pre-participation survey (YAD)	Post-participation survey (YAD)	Post-participation survey (DEs)	Focus group (DEs)
(a) Efficacy	Validated psychometric instruments, HbA1c	Validated psychometric instruments, HbA1c		
(b) Acceptability		Responses to open-ended comments	Responses to open-ended comments	Qualitative data

### Data sources

All online surveys were hosted on Qualtrics, an online survey platform.^26^

*Pre- and post-participation surveys (YAD)*. YAD was invited to participate in surveys before they commenced the GSD program and three months after their last conversation with the DE. By express consent, surveys were not anonymous, as matching of pre- and post-participation surveys was required.

The pre-participation surveys assessed YAD demographic and health characteristics (participants’ sex, living arrangements, years since diabetes diagnosis, diabetes complications, and current medications for diabetes).

The following validated psychometric instruments were included in the pre- and post-participation surveys for YAD:

*Perceived Competence for Diabetes Scale (PCS) (four items):* Assesses perceived competence in managing one's diabetes. Competence is assumed to be one of three fundamental psychological needs, so feelings or perceptions of competence with respect to diabetes are important because they facilitate people's goal attainment.^27^ Responses for each item, assessed on a Likert Scale ranging from 1 (not at all true) to 7 (very true); total scores are presented as the mean of ratings on the four items. Higher scores indicate optimal competence in managing diabetes.

*Treatment Self-regulation Scale (TSRQ) (19 items):* Assesses a person's motivation for engaging in health behaviours, namely taking medications, self-checking blood glucose, following an appropriate eating program and exercising regularly.^9^ Specifically, the scale measures the degree to which a person's motivation is autonomous (Autonomous motivation subscale, 11 items) or the respondent is motivated by expectations of other significant people in their life (Controlled motivation subscale, 8 items). Autonomous motivation is a key principle of self-determination theory, which underpins the GSD approach.^12^ Responses for each item, assessed on a Likert Scale ranging from 1 (not at all true) to 7 (very true); total scores are calculated as the mean of ratings for each subscale.^14^ Higher scores indicate optimal motivation for the Autonomous motivation subscale, while lower scores indicate optimal motivation for the Controlled subscale.

*Health Care Climate Questionnaire (HCCQ) (15 items):* Assesses clients’ perceptions of the degree to which their health care provider, in this case, the participating DEs facilitating the online GSD program, is supportive of their autonomous decision-making.^28^ Sample items include ‘I feel that my DE has provided me choices and options’ and ‘My DE tries to understand how I see things before suggesting a new way to do things’. Each item was rated on a 7-point Likert scale ranging from 1 (strongly disagree) to 7 (strongly agree). Total scores are calculated as the mean of ratings on the 15 items. Higher scores indicate optimal healthcare environment.

*HbA1c:* Respondents were asked to provide their most recent blood HbA1c levels

To assess acceptability of the program from YAD’ perspectives, the post-participation survey included 7 Likert-scale questions assessing their perceptions of the GSD website and Zoom/ telephone communication with DEs, and four open-ended questions inviting free-text comments

*Post-participation surveys (DEs).* To assess acceptability of the program from DEs’ perspectives, anonymous evaluation surveys were distributed to DEs on completion of the GSD program with at least two YAD. These surveys assessed demographic and work characteristics (participants’ age, qualification, and years of experience as a DE). To assess acceptability of the program from DEs’ perspectives, four Likert scale questions assessed DEs’ perspectives on the GSD approach, five Likert-scale questions assess their perspectives of the GSD website and an open-ended question invited any other comments about the GSD program

*Focus group (DEs).* Following completion of the GSD program with at least two YAD, DEs were also invited to a focus group hosted on Zoom. The focus group was facilitated by BR and KW.

### Data analysis

Quantitative data were analysed using IBM SPSS Statistics v25. Scores for the PCS, TSRQ, and HCCQ were calculated according to the instructions of each instrument's original authors. Scores on these scales and client-reported blood glucose levels (pre- and post-GSD program completion) were not normally distributed; therefore, the non-parametric Wilcoxon Signed Ranks test was used to test for differences within individuals across time. Significance was assessed at *p* < 0.05. Medians are presented, together with means (SD) which are reported for ease of interpretation only.

Responses to open-ended survey questions were managed using an Excel spreadsheet and a manual text analysis^29^ was conducted with a broad-brush line-by-line coding, and cross checked by two researchers (BR, KW).

Focus group data were analysed from notes obtained by the facilitators and a manual thematic analysis was applied, using a modified version of Braun and Clarke^30^ analysis method: (1) familiarisation with the data, (2) searching for patterns or themes, and (3) reviewing and comparing facilitators’ notes and four reporting themes.

### Ethical considerations

The study was approved by the ethics committee of Western Health (Ethics ID HREC/18/WH/1130) and conducted according to the Declaration of Helsinki. Plain language statements included assurance that if YAD and DEs withdrew from the study it would not affect their care, and that confidentiality of information, privacy and dignity would be observed throughout the study.

## Results

Nine DEs were recruited and trained. Three mentors, one from the research team, experienced in using the GSD program, also attended the training to provide supervision and support. Eight DEs completed the GSD program with at least one young adult. Of these, four completed it with two YAD and one with three YAD. Six diabetes educators (with 7–14 years of experience working as a diabetes educator) completed the post-participation survey. Five DEs responded to email invitation and attended the focus group.

Forty YAD consented to participate. Of these, 18 (19–30 years) commenced the GSD program with a trained DE; the remaining 22 YAD could not be contacted after they provided consent, or in discussion with researchers indicated that they did not wish to proceed with the program owing to employment, study or family commitments. Of the 18 who commenced the program, 13 (72%) completed the program.

Pre- and post-participation survey data were available for eight YAD; the remaining five participants did not complete the post-participation survey. Of those for whom complete data were available, seven were female and one was male. Four respondents indicated that they lived with their parents, three with their partner and one with other people. Time since diagnosis with T1D ranged from 2.5 to 17 years; five used an insulin pump. No participants reported any diabetes-related complications.

### Efficacy of the online GSD program to improve self-management of T1D among YAD

There was a significant improvement in respondents’ scores for the autonomous motivation subscale of the TSRQ (*p* = 0.028) and the HCCQ scale (*p* = 0.046). There was no significant difference in PCS scores, TSRQ controlled motivation subscale scores or self-reported HbA1c, following completion of the online GSD program (Table 2).

**Table 2.** Young adults’ pre- and post-participation scores on validated measures and self-reported HbA1C.

*Scale* ^a^	*Pre-participation*		Post-participation		*p* ^b^
	*Median*	Mean (SD)	Median	Mean (SD)	
*Perceived Competence Scale (PCS): Diabetes*	*5*.*38*	5.25 (1.19)	6.50	6.00 (0.96)	0.126
Treatment Self-Regulation Scale Questionnaire (TSRQ): Autonomous motivation	5.00	4.70 (1.24)	5.69	5.31 (1.01)	0.028
Treatment Self-Regulation Questionnaire (TSRQ): Controlled motivation	3.57	3.41 (1.21)	3.82	3.66 (0.60)	0.482
Health Care Climate questionnaire (HCCQ)	4.97	4.73 (1.18)	6.43	6.28 (0.40)	0.046
Self-reported HbA1c	7.40	7.49 (1.01)	7.70	7.58 (1.41)	1.00

^a^ Possible range of scores 1–7 on each scale. Higher scores more optimal, except for the treatment self-regulation questionnaire: controlled motivation, for which lower scores are optimal.

^b^ Significance value associated with Wilcoxon Signed Ranks test for differences within related samples.

### Acceptability of the online GSD program from the perspectives of YAD and DEs

*Young adults with type 1 diabetes.* Respondents’ comments highlighted an overall high satisfaction with the program, including the ability to ask questions usually not asked by health professionals. YAD indicated they did not feel judged for asking questions and gained a deeper understanding of their diabetes. For a couple of YAD ‘it changed their lives’. The YAD also highlighted challenges experienced in program participation such as questions were repetitive, or not specifically relevant to their situation; the website's user interface and navigation could be smoother to operate; one gave suggestions for how it could be done.

Responses to Likert-scale items assessing young adults’ perceptions and experiences of the GSD program (post-participation survey), showed agreement that the GSD website was easy to use, the website layout was user friendly, and that the reflection boxes in the GSD conversations were useful. Seven of eight respondents found navigation between conversations on the GSD website easy; the remaining participant responded, ‘neither agree nor disagree’. All respondents agreed that the teleconferencing program (e.g., Zoom) utilised for conversations with DEs was easy to use and adequate for communicating with their DE. One participant indicated they would have preferred in-person (rather than online) conversations with their DE; three disagreed, one selected ‘neither agree nor disagree’ and one did not respond to this question.

Examples of young adult participants’ responses to open-ended comments are provided in Supplement 4.

*Diabetes Educators.* Responses to Likert-scale items assessing DEs’ perceptions and experiences of the GSD program (post-participation survey) indicated unanimous agreement that the GSD method is an effective approach for working with YAD to improve their self-management of diabetes. Respondents would continue to use aspects of the GSD method with young adults as well as other clients with T1D, and they would recommend the GSD approach to other diabetes educators. Regarding the online GSD platform, 4 of 6 respondents indicated that they found the platform easy to use, and their YADs expressed the same view. Five respondents indicated that if they had open ongoing access to the platform, they would use it with YAD, and four said they would use it with other adult clients with T1D. Five indicated that they would recommend the online GSD program to other DEs.

Free-text comments by DE respondents indicated they valued the GSD program, including that it was a useful tool to guide conversations. Keeping track of progress was a very valuable and rewarding experience. However, DEs highlighted there were challenges, mainly related to recruitment difficulties.

Examples of free-text comments written by the DE survey respondents are provided in Supplement 5.

Overall, for the DEs who attended the focus group, their experience of facilitating the GSD program with YAD was positive, particularly as it related to ways to engage with young people and their own professional growth. DEs highlighted that they learnt to improve their communication skills, such as using pauses and active listening skills. Time was an important factor in terms of YAD motivation because YAD needed time to reflect on their issues before they could decide on solutions.

Focus group participants indicated the main challenges related to getting the YAD involved in the program in the first instance and technological issues, were the user experience and expecting smoother navigation once on the website.

DEs emphasised that some text in the online activities required high literacy skills of YAD and therefore suggested using tools to improve accessibility for those with lower health literacy skills. Examples of the DEs responses to the specific focus group question are illuminated in Supplement 6.

## Discussion

### Communication and motivation experiences in GSD

*Young adults.* YAD found that GSD online enhanced the communication with their DEs as their conversations were more individualised, and solution orientated. These findings echo findings from our previous pilot study in which YAD also highlighted that the option of accessing the GSD platform as required was considered essential to their reflection and decision-making.^20^

In the current study, the GSD online program captured YAD information that may otherwise have not been shared with DEs and was used to inform management strategies that helped YAD see patterns of behaviours, recurring issues or difficulties in diabetes self-management. Zoffmann and Kirkevold reported that by completing GSD reflections, clients improved their ability to identify, express, and share unique and unexpected difficulties related to living with diabetes.^13^ As signs of empowerment, YAD and DEs reported shared decision-making which helped to establish meaningful and effective relationships^13^ enhancing a person-centred approach in self-management of diabetes.^21^ Zoffmann and Kirkevold^13^ highlighted that to keep motivated and feel empowered, YAD needs to understand their own roles in these barriers and enablers.^16^ In current study, the YAD reported that during the GSD program, they talked to their DEs about issues in their lives that were most pertinent at the time they got involved in the GSD program, not their diabetes. They indicated that because the conversations were centred on their terms and needs, YAD was able to understand their diabetes in a wider context and take responsibility for their own care to keep themselves motivated. Similar findings have been reported that indicate using GSD seems to positively influence motivation for self-management.^31^ The same study reported that through self-reflection about how to live with diabetes, participants reinterpreted their life with diabetes by gradually moving toward acceptance of their condition because the dialogue with the DEs was seen to be on a more ‘equal footing’ compared to usual consultations, which, in turn, helped support participants to become more self-determined.^31^ YAD in this study highlighted that time to reflect and having open conversations helped them to stay motivated.

*Diabetes Educators.* DEs found that the GSD program had changed their practice in the way they communicated with YAD. DEs listened and reflected more when using GSD structured conversations. Thus, DEs implemented more of a shared decision-making and person-centred approach with YAD. These changes resulted in a higher level of engagement, motivation and satisfaction in working with the YAD as they observed the YAD become more self-sufficient and motivated.

Thus, online GSD provided educational and behavioural support for DEs and YAD. A Danish qualitative study investigated nurses’ experiences of learning and using the GSD method in gynaecological settings to determine whether the GSD method influenced nurses’ professional practice.^29^ Most nurses indicated that GSD was a supplement to their practice and professional role because they gained new person-centred knowledge about client challenges. They reported a higher level of confidence in communicating with YAD and more satisfaction in their work.^29^

DEs also reported that feeling confident was important in facilitating the online GSD method, including their technology skills. Therefore, appropriate training and support for DEs are essential in program implementation. Training health professionals in digital technology is well recognised in both international and national literature,^30,31^ especially in the COVID-19 pandemic environment with the expansion of use of eHealth systems and increased focus on supporting consumers to directly engage with and use online health care services.^32^

### Online platform experience

The overall experience of undertaking the GSD program using the online platform was positive from both YAD’ and DEs’ perspectives. Despite a few technical shortcomings, the interactive conversations encouraged reflections and enhanced meaningful communication which assisted YAD to self-manage diabetes and their lives and DEs to apply new communication skill to their practice; essential to deliver effective person-centred self-management support.^32^ To provide meaningful communication among health professionals, it is essential that the technology meets the end users’ needs.^33,34^ Researchers who have addressed models of building capabilities using technology indicate that it is integral for users to believe the technology is beneficial, engaging and manageable.^35^ The flexibility of the online GSD and engagement between the participants were found to the most important aspects of the online experience of both YAD and DEs, especially it helped YAD to balance competing commitments between work, studies and families, which otherwise may have been difficult for example to fit medical appointments into their busy schedules. It is well established in the literature that online consultations are particularly attractive to this age group of young adults as they save time, expenses related to transport and taking time off work.^36–38^ These three issues have been established as major barriers preventing young adults with T1D aged 18–30 years accessing health services.^39^ The technology and communication flexibility offered by online GSD helped DEs establish relationships with YADs faster than face-to-face diabetes consultations which, in turn, provided practical solutions and reassurance to YADs in a timely manner that kept motivation and confidence high for YAD managing their lives with diabetes.^21^ The GSD conversations are therefore in line with the evidence, and the ready accessibility and interactive conversations in the platform may have supported YAD to stay motivated.

### Study strengths and limitations

Strengths of this study include the use of validated psychometric instruments to assess changes in YAD self-management motivation, self-regulation and communication with DEs. The mixed-methods approach enabled rich data to be gathered regarding acceptability and feasibility of the online GSD program from the perspectives of both YAD and DEs to be captured.

Limitations include the small sample size. Recruitment of YAD proved very difficult. YAD cited issues related to time pressures and balancing competing work, study and family commitments, preventing their participation, although their reluctance to participate may also indicate a low priority placed on DM management at this stage of their lives, and this was probably reflected in the number of YAD who consented but did not engage in the GSD program. Data was not collected about the quality of internet access for YAD and this may have been an issue in large households and in rural areas with poor internet access at the time this study was undertaken.

**Figure 2.**
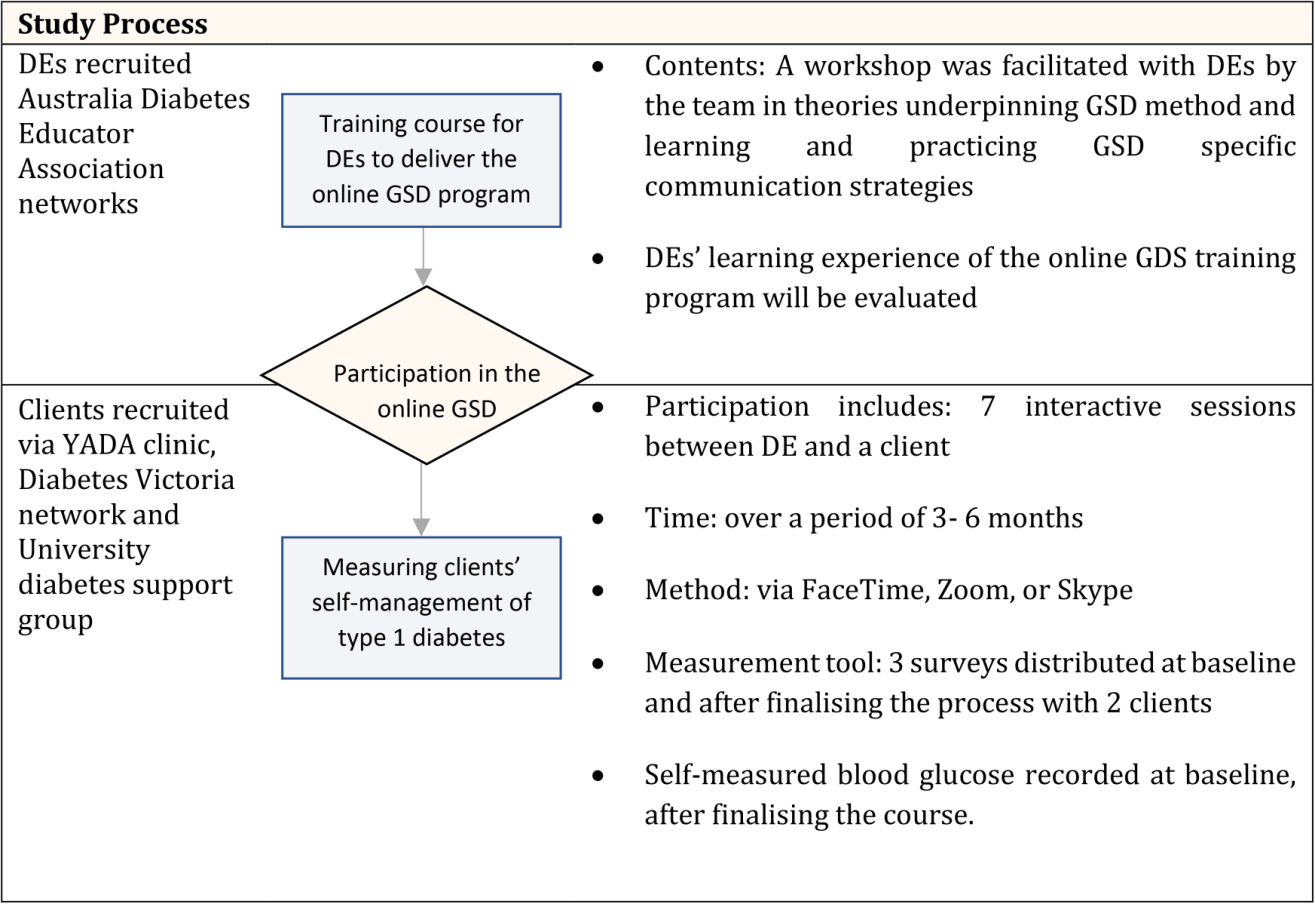
Study design, process and procedures.

*Note:* GSD: Guided Self-Determination. DE: Diabetes Educator.

It is possible that the sample was not representative of YAD because participants may have been more technologically competent and confident and had reasonable health literacy skills than those who did not participate.

## Conclusion

This study confirms that an online GSD program had a statistically significant impact on autonomous motivation in the diabetes self-management of those YAD who participated in this program. The online version is feasible and acceptable in assisting YAD to communicate with DEs about pertinent diabetes concerns and identify appropriate solutions. With further evaluation of those YAD who did not participate in the program, potentially the program might be more widely applicable. DEs appreciated the flexibility of the online GSD program and reported that it provided positive opportunities to engage and communicate with YAD, hence enhancing their practice. Our evaluation of this online GSD program demonstrated a reproducible model of facilitation of diabetes self-management for YAD. For sustainability, the GSD programs should be integrated as part of treatment-as-usual in health services and in DEs’ own practice. Future evaluations should be based on longitudinal and multicentred programs.

## Practice implications

Communication technologies such as the online Guided Self-determination platform used in this study contribute to effective diabetes self-management due to their accessibility and relevance to end users. Online GSD also provides health professionals such as DEs opportunities to improve engagement and communication with their clients.

The online guided self-determination program contributes to person-centred, developmentally age-appropriate care delivery by supporting YAD to be motivated and engaged with managing life with diabetes.

The online platform offers potential opportunities to reach populations that otherwise can be hard to reach due to geographical distances, social circumstances and other barriers which impede face-to-face health service delivery.

Further research about training of health professionals in using technology is essential to improved health care delivery in diabetes care.

## Supplemental Material

sj-docx-1-dhj-10.1177_20552076231167008 - Supplemental material for Feasibility and acceptability of an online guided self-determination program to improve diabetes self-management in young adults Click here for additional data file. Supplemental material, sj-docx-1-dhj-10.1177_20552076231167008 for Feasibility and acceptability of an online guided self-determination program to improve diabetes self-management in young adults by Bodil Rasmussen, Karen Wynter, Peter S Hamblin, Christine Rodda, Cheryl Steele, Sara Holton, Vibeke Zoffmann and Judy Currey in Digital Health

sj-docx-2-dhj-10.1177_20552076231167008 - Supplemental material for Feasibility and acceptability of an online guided self-determination program to improve diabetes self-management in young adults Click here for additional data file. Supplemental material, sj-docx-2-dhj-10.1177_20552076231167008 for Feasibility and acceptability of an online guided self-determination program to improve diabetes self-management in young adults by Bodil Rasmussen, Karen Wynter, Peter S Hamblin, Christine Rodda, Cheryl Steele, Sara Holton, Vibeke Zoffmann and Judy Currey in Digital Health

sj-docx-3-dhj-10.1177_20552076231167008 - Supplemental material for Feasibility and acceptability of an online guided self-determination program to improve diabetes self-management in young adults Click here for additional data file. Supplemental material, sj-docx-3-dhj-10.1177_20552076231167008 for Feasibility and acceptability of an online guided self-determination program to improve diabetes self-management in young adults by Bodil Rasmussen, Karen Wynter, Peter S Hamblin, Christine Rodda, Cheryl Steele, Sara Holton, Vibeke Zoffmann and Judy Currey in Digital Health

sj-docx-4-dhj-10.1177_20552076231167008 - Supplemental material for Feasibility and acceptability of an online guided self-determination program to improve diabetes self-management in young adults Click here for additional data file. Supplemental material, sj-docx-4-dhj-10.1177_20552076231167008 for Feasibility and acceptability of an online guided self-determination program to improve diabetes self-management in young adults by Bodil Rasmussen, Karen Wynter, Peter S Hamblin, Christine Rodda, Cheryl Steele, Sara Holton, Vibeke Zoffmann and Judy Currey in Digital Health

sj-docx-5-dhj-10.1177_20552076231167008 - Supplemental material for Feasibility and acceptability of an online guided self-determination program to improve diabetes self-management in young adults Click here for additional data file. Supplemental material, sj-docx-5-dhj-10.1177_20552076231167008 for Feasibility and acceptability of an online guided self-determination program to improve diabetes self-management in young adults by Bodil Rasmussen, Karen Wynter, Peter S Hamblin, Christine Rodda, Cheryl Steele, Sara Holton, Vibeke Zoffmann and Judy Currey in Digital Health

sj-docx-6-dhj-10.1177_20552076231167008 - Supplemental material for Feasibility and acceptability of an online guided self-determination program to improve diabetes self-management in young adults Click here for additional data file. Supplemental material, sj-docx-6-dhj-10.1177_20552076231167008 for Feasibility and acceptability of an online guided self-determination program to improve diabetes self-management in young adults by Bodil Rasmussen, Karen Wynter, Peter S Hamblin, Christine Rodda, Cheryl Steele, Sara Holton, Vibeke Zoffmann and Judy Currey in Digital Health
