# Supplementary material for: Feasibility and acceptability of an online guided self-determination program to improve diabetes self-management in young adults
Source: Digit Health. 2023 Mar 30;9:20552076231167008. doi: 10.1177/20552076231167008 (PMC10068990; doi:10.1177/20552076231167008)
Supplement: sj-docx-1-dhj-10.1177_20552076231167008 - Supplemental material for Feasibility and acceptability of an online guided self-determination program to improve diabetes self-management in young adults [file sj-docx-1-dhj-10.1177_20552076231167008.docx]

**Screening question**

*How old are you? (in years)

- Younger than 18 (skip to “Unfortunately you are not eligible to take part in this study, which is only for young adults aged 18 to 30. Thanks for your interest!)
- 18 - 30
- Older than 30 (skip to “Unfortunately you are not eligible to take part in this study, which is only for young adults aged 18 to 30. Thanks for your interest!)

**About you and your diabetes**

*Please indicate your sex:

- Female
- Male
- Other (please write) ________________________________________________

*What is your postcode? _____________________________________________

*Please indicate your current living arrangements (select all that apply):

- I live on my own
- I live with my parents
- I live with my sibling/s (but not my parents)
- I live with a partner
- I live with other people (not parents, siblings, partner)
- Other, please specify _______________________________________________

*How long is it since you were diagnosed with diabetes? (in years)

________________________________________________________________

|  |
| --- |

*Which health professionals currently provide you with information and support in regards to your diabetes? (eg GP, diabetes educator)

________________________________________________________________

Please list the medications you are currently taking, the doses and how often you take them.

________________________________________________________________

Do you have an insulin pump?

- No
- Yes

In an average month, how often to do find yourself hypoglycaemic (<4mmol/L)?

________________________________________________________________

In an average month, how often to do find yourself hyperglycaemic (>10mmol/L)?

________________________________________________________________

Please list any diabetes-related complications:

________________________________________________________________

Please provide the approximate date and results of your most recent HbA1c test:

________________________________________________________________

**Perceived Competence Scale**

Please respond to each of the following items in terms of how true it is for you with respect to dealing with your diabetes.

|  | Not at all true |  |  | Somewhat true |  |  | Very true |
| --- | --- | --- | --- | --- | --- | --- | --- |
|  | 1 | 2 | 3 | 4 | 5 | 6 | 7 |
| I feel confident in my ability to manage my diabetes. |  |  |  |  |  |  |  |
| I am capable of handling my diabetes now. |  |  |  |  |  |  |  |
| I am able to do my own routine diabetic care now. |  |  |  |  |  |  |  |
| I feel able to meet the challenge of controlling my diabetes. |  |  |  |  |  |  |  |
| I feel confident in my ability to learn this learning material. |  |  |  |  |  |  |  |
| I am capable of learning the material in this program. |  |  |  |  |  |  |  |
| I am able to achieve my goals in this course. |  |  |  |  |  |  |  |
| I feel able to meet the challenge of performing well in this program. |  |  |  |  |  |  |  |

**Treatment Self-Regulation Questionnaire**

Please consider the following behaviours and indicate how true each of these is for you.

I take my medications for diabetes and/or check my glucose because:

|  | Not at all true |  |  | Somewhat true |  |  | Very true |
| --- | --- | --- | --- | --- | --- | --- | --- |
|  | 1 | 2 | 3 | 4 | 5 | 6 | 7 |
| Other people would be angry with me if I didn't. |  |  |  |  |  |  |  |
| I find it a personal challenge to do so. |  |  |  |  |  |  |  |
| I personally believe that controlling my diabetes will improve my health. |  |  |  |  |  |  |  |
| I would feel guilty if I didn't do what my doctor said. |  |  |  |  |  |  |  |
| I want my doctor to think I'm a good patient. |  |  |  |  |  |  |  |
| I would feel bad about myself if I didn't. |  |  |  |  |  |  |  |
| It's exciting to try to keep my glucose in a healthy range. |  |  |  |  |  |  |  |
| I don't want other people to be disappointed in me. |  |  |  |  |  |  |  |

The reason I follow my diet and exercise regularly is that:

|  | Not at all true |  |  | Somewhat true |  |  | Very true |
| --- | --- | --- | --- | --- | --- | --- | --- |
|  | 1 | 2 | 3 | 4 | 5 | 6 | 7 |
| Other people would be upset with me if I didn't. |  |  |  |  |  |  |  |
| I personally believe that these are important in remaining healthy. |  |  |  |  |  |  |  |
| I would be ashamed of myself if I didn't. |  |  |  |  |  |  |  |
| It is easier to do what I'm told than to think about it. |  |  |  |  |  |  |  |
| I've carefully thought about my diet and exercising and believe it's the right thing to do. |  |  |  |  |  |  |  |
| I want others to see that I can follow my diet and stay fit. |  |  |  |  |  |  |  |
| I just do it because my doctor said to. |  |  |  |  |  |  |  |
| I feel personally that watching my diet and exercising are the best things for me. |  |  |  |  |  |  |  |
| I'd feel guilty if I didn't watch my diet and exercise. |  |  |  |  |  |  |  |
| Exercising regularly and following my diet are choices I really want to make. |  |  |  |  |  |  |  |
| It's a challenge to learn how to live with diabetes. |  |  |  |  |  |  |  |

**Health-Care Climate Questionnaire**

This questionnaire contains items that are related to your visits with your diabetes educator. Diabetes educators have different styles in dealing with patients, and we would like to know more about how you have felt about your encounters with your diabetes educator. Your responses are confidential. Please be honest and candid. Note: If you don't currently have any contact with a diabetes educator, please choose "Not applicable".

|  | Not at all true |  |  | Somewhat true |  |  | Very true |
| --- | --- | --- | --- | --- | --- | --- | --- |
|  | 1 | 2 | 3 | 4 | 5 | 6 | 7 |
| I feel that my diabetes educator has provided me choices and options. |  |  |  |  |  |  |  |
| I feel understood by my diabetes educator |  |  |  |  |  |  |  |
| I am able to be open with my diabetes educator at our meetings. |  |  |  |  |  |  |  |
| My diabetes educator conveys confidence in my ability to make changes. |  |  |  |  |  |  |  |
| I feel that my diabetes educator accepts me. |  |  |  |  |  |  |  |
| My diabetes educator has made sure I really understand about my condition and what I need to do. |  |  |  |  |  |  |  |
| My diabetes educator encourages me to ask questions. |  |  |  |  |  |  |  |
| I feel a lot of trust in my diabetes educator. |  |  |  |  |  |  |  |
| My diabetes educator answers my questions fully and carefully. |  |  |  |  |  |  |  |
| My diabetes educator answers my questions fully and carefully. |  |  |  |  |  |  |  |
| My diabetes educator listens to how I would like to do things |  |  |  |  |  |  |  |
| My diabetes educator handles people's emotions very well. |  |  |  |  |  |  |  |
| I feel that my diabetes educator cares about me as a person. |  |  |  |  |  |  |  |
| I don't feel very good about the way my diabetes educator talks to me. |  |  |  |  |  |  |  |
| My diabetes educator tries to understand how I see things before suggesting a new way to do things. |  |  |  |  |  |  |  |
| I feel able to share my feelings with my diabetes educator. |  |  |  |  |  |  |  |

** How did you meet with your diabetes educator?

- Zoom
- Facetime
- Other, please specify ________________________________________________

** Please indicate the extent to which you agree with each of the following statements:

|  | Strongly disagree | Disagree | Somewhat disagree | Neither agree nor disagree | Somewhat agree | Agree | Strongly agree | Not applicable / did not use |
| --- | --- | --- | --- | --- | --- | --- | --- | --- |
| The GSD website was easy for me to use |  |  |  |  |  |  |  |  |
| The layout of the GSD website was user friendly |  |  |  |  |  |  |  |  |
| The reflection boxes in the GSD conversations were useful to me |  |  |  |  |  |  |  |  |
| It was easy to navigate between the conversations on the GSD website |  |  |  |  |  |  |  |  |
| The teleconferencing program (eg Zoom) was easy for me to use |  |  |  |  |  |  |  |  |
| The teleconferencing program (eg Zoom) was adequate for communicating with the diabetes educator |  |  |  |  |  |  |  |  |
| I would have preferred face-to-face (rather than online) conversations with a diabetes educator |  |  |  |  |  |  |  |  |

** (If participant indicated disagreement with any of the above statements) You have indicated that your experience of the GSD website could have been better. Please suggest how you would improve the GSD website:

________________________________________________________________

** What did you like most about the GSD program?

________________________________________________________________

** What would you change about the GSD program?

________________________________________________________________

** Please add any other comments about the GSD program.

________________________________________________________________

*Included in the pre-participation survey only

** Included in the post-participation survey only
