# Supplementary material for: Feasibility and acceptability of an online guided self-determination program to improve diabetes self-management in young adults
Source: Digit Health. 2023 Mar 30;9:20552076231167008. doi: 10.1177/20552076231167008 (PMC10068990; doi:10.1177/20552076231167008)
Supplement: sj-docx-2-dhj-10.1177_20552076231167008 - Supplemental material for Feasibility and acceptability of an online guided self-determination program to improve diabetes self-management in young adults [file sj-docx-2-dhj-10.1177_20552076231167008.docx]

**Some questions about you**

What was your age at your last birthday? ______________

What is your residential postcode? _____________________

What are your qualifications? _____________________

How long have you worked as a Diabetes Educator (in years)? _____________________

**Your experiences of GSD**

Please indicate the extent to which you agree with each of the following statements about the overall GSD approach:

|  | Strongly disagree | Disagree | Agree | Strongly agree | Don’t know / prefer not to answer |
| --- | --- | --- | --- | --- | --- |
| The GSD method is an effective approach for working with young adults with diabetes |  |  |  |  |  |
| In the future, I will continue to use aspects of the overall GSD approach with young adults with type 1 diabetes |  |  |  |  |  |
| In the future, I will use aspects of the overall GSD approach with other clients with type 1 diabetes |  |  |  |  |  |
| I would recommend the overall GSD approach to other diabetes educators |  |  |  |  |  |

Please indicate the extent to which you agree with each of the following statements about the GSD online platform:

|  | Strongly disagree | Disagree | Agree | Strongly agree | Don’t know / prefer not to answer |
| --- | --- | --- | --- | --- | --- |
| I found the GSD online platform easy to use |  |  |  |  |  |
| The young adults I worked with found the GSD online platform easy to use |  |  |  |  |  |
| If I had access to the online GSD platform, I would use it with young adult clients with type 1 diabetes |  |  |  |  |  |
| If I had access to the online GSD platform, I would use it with other clients with type 1 diabetes |  |  |  |  |  |
| I would recommend the online GSD program to other diabetes educators |  |  |  |  |  |

Please share any other comments about your experiences with the GSD approach, the online platform or this research project.

________________________________________________________________
