# Supplementary material for: Feasibility and acceptability of an online guided self-determination program to improve diabetes self-management in young adults
Source: Digit Health. 2023 Mar 30;9:20552076231167008. doi: 10.1177/20552076231167008 (PMC10068990; doi:10.1177/20552076231167008)
Supplement: sj-docx-3-dhj-10.1177_20552076231167008 - Supplemental material for Feasibility and acceptability of an online guided self-determination program to improve diabetes self-management in young adults [file sj-docx-3-dhj-10.1177_20552076231167008.docx]

**A discussion guide was developed including the following questions:**

1. What are your thoughts about GSD an approach for working with young adults with diabetes?

2. GSD was specifically designed to improve self-management among people with diabetes, who have lost motivation. What are your thoughts about this?

3. What was your experience of doing the GSD *online* program (advantages / disadvantages)?

4. What changes do you suggest to improve the experience of the online GSD program (for clients / for DEs).

5. Please share any other experiences of your journey with GSD.
