# Supplementary material for: Feasibility and acceptability of an online guided self-determination program to improve diabetes self-management in young adults
Source: Digit Health. 2023 Mar 30;9:20552076231167008. doi: 10.1177/20552076231167008 (PMC10068990; doi:10.1177/20552076231167008)
Supplement: sj-docx-4-dhj-10.1177_20552076231167008 - Supplemental material for Feasibility and acceptability of an online guided self-determination program to improve diabetes self-management in young adults [file sj-docx-4-dhj-10.1177_20552076231167008.docx]

**Supplement 4: Illustrative examples from YAD’ responses to open-ended survey questions**

| **Open-ended question** | **Responses from YAD** |
| --- | --- |
| What did you like most about the GSD program? | *Being able to talk about different challenges to diabetes that you don't tend to talk about with the endocrinologist.*  *I was able to understand my diabetes and problems thoroughly*  *The open/candid conversations*  *That it was flexible*  *How it allowed me to be honest about my struggles and not feel shame. Made me feel very supported and gave me so many options to control my BGLs I didn't know I had*  *It gave me a chance to reflect on my chronic condition management*  *The conversations and questions (the problem-solving method) were great tools that I will genuinely use again and again* |
| What would you change about the GSD program? | *Nothing*  *Some of the questions seemed repetitive*  *Some of the questions we had to answer for the program were not super relevant, I feel that they could be a bit more realistic/easier to answer*  *Would have it for longer but with less contact hours after the initial months, maybe a follow up session and review of my goals*  *Make the wording more friendly to groups with lower health literacy levels*  *It would be handy to get a summary or bundle of all the conversations in an email/document so it is easier to bring along to my endo/GP appointments* |
| Please add any other comments about the GSD program. | *In all I think it's really good in being able to talk/rant about diabetes but also come up with strategies to help combat those challenges!*  *I'm glad I received the opportunity to participate as it changed my life :)*  *It was a great experience*  *Thank you* |
| You have indicated that your experience of the GSD website could have been better. Please suggest how you would improve the GSD website. | *Some of the answer boxes were sometimes difficult to access/didn't work*  *Sometimes the buttons etc did not take you to the right place, and one week had the last weeks information*  *The user interface needs a bit of work at times because the questions can be confusing but overall gets the job done. Navigating it along without the diabetes educator would have been more challenging.*  *The website was a bit clunky in some areas where the questions would be cut off (on Apple iPad). It would be really handy to have a progress side bar or banner to see what conversation you are up to and how much more to go without having to go back to the conversation page* |
