# Supplementary material for: Feasibility and acceptability of an online guided self-determination program to improve diabetes self-management in young adults
Source: Digit Health. 2023 Mar 30;9:20552076231167008. doi: 10.1177/20552076231167008 (PMC10068990; doi:10.1177/20552076231167008)
Supplement: sj-docx-5-dhj-10.1177_20552076231167008 - Supplemental material for Feasibility and acceptability of an online guided self-determination program to improve diabetes self-management in young adults [file sj-docx-5-dhj-10.1177_20552076231167008.docx]

**Supplement 5: Illustrative examples from DEs’ responses to open-ended survey questions**

| **Open-ended question** | **DEs’ Responses** |
| --- | --- |
| Based on your experience of using GSD, name at least one way in which your communication with your clients in general has changed | *I ask a lot more questions with the intention to give the client the feeling that they are in control*  *The focus on the client's experience. - Giving more space and time for the client to express their perception and perspective throughout the process.*  *Remembering to pause to allow time for the patient to think and respond. Revisiting the 3 types of communication to help the patient identify the key issues. Also, re-looking at ways to address a problem in a different way, help them reflect on what has been happening- things to work on and things going well.*  *Open conversations, less pre-determined appointments, open to look at other things that impacts on self-care*  *I pause more to allow more time for clients to think and respond*  *In response to Active listening and mirroring has been excellent to begin the conversation.* |
| Based on your experience of doing GSD with your clients, name at least one way in which you think the YAD you worked with may have changed the way they self-manage their diabetes or approach to living with diabetes | *That they are now making the decisions about how they deal with their diabetes. They feel more in control*  *With both clients there was a shift from "I should do" to "this is how I see the challenge"*  *Looking at challenges with a different perspective. Remembering to reflect back. Also breaking down a challenge and working practically with what they can do.*  *I have more open thoughts, be less strung up about the expectations from other HCP, be more actively involved with HCP appointments to meet their own needs, not trying to 'fix/solve' everything all at once*  *Think outside the box, I would hope the client would reflect back on the steps in the process* |
| Please share any other comments about your experiences with the GSD approach, the online platform or this research project. | *It was a useful tool to structure and guide the conversations which aided keeping things on track*  *Where shall I start! Thank you! For myself it was a very valuable and rewarding experience that has taught me a lot*  *I found the participants’ lack of commitment frustrating because they had volunteered to be in the program*  *It has been a steep, worthwhile learning curve. Putting theory into practice with YAD was challenging and encouraged me to critically review my interactions* |
