# Supplementary material for: Feasibility and acceptability of an online guided self-determination program to improve diabetes self-management in young adults
Source: Digit Health. 2023 Mar 30;9:20552076231167008. doi: 10.1177/20552076231167008 (PMC10068990; doi:10.1177/20552076231167008)
Supplement: sj-docx-6-dhj-10.1177_20552076231167008 - Supplemental material for Feasibility and acceptability of an online guided self-determination program to improve diabetes self-management in young adults [file sj-docx-6-dhj-10.1177_20552076231167008.docx]

**Supplement 6: Illustrative quotations representing DEs’ responses to focus group questions**

| **Open-ended question** | **DEs’ Responses** |
| --- | --- |
| What are your thoughts about GSD an approach for working with YAD? | *Changed way of doing things, benefited personally (realised when doing assessment)*  *Course team’s communication activities – have learned so much*  *It was tedious to have to chase young people, if the young person wanted to do everything with diabetes educator it was not practical, it is better to go with their flow-and set tasks to save time and there is a need for setting a time frame*  *Seemed to be motivated when they realised it was winding up.*  *In real practice, I can’t be chasing up or [working with someone] who is not turning up*  *Program was great.*  *One young adult became very self-sufficient and was aware of this after 4 months of silence looked at diabetes differently.*  *In the beginning I was negative and scared to say anything wrong then became positive*  *It was difficult to get young people into the program. I tried 3 people [3 strikes]*  *It was beneficial to talk to team member [ before going into] conversations [as it] gave confidence.*  *Conversations present opportunity to say: what else?* |
| How about GSD online? | *Regarding the platform: it was not ideal as we had to go back to home page every time between conversations*  *Platform very smooth*  *Standard of language [was high literacy], may be assess using tools for health literacy and readability*  *Questions sometimes negatively worded – not YAD have problems especially in the unfinished sentences where language a little negative. The purpose is to established good qualities in life approach and approach to diabetes*  *Offering no camera – in case they [young person] are uncomfortable* |
